# Empathy and the art of Leonardo da Vinci

**DOI:** 10.3389/fpsyg.2023.1260814

**Published:** 2024-03-08

**Authors:** Samira Schultz Mansur, Javier DeFelipe

**Affiliations:** ^1^Department of Morphological Sciences, Biological Science Center, Federal University of Santa Catarina, Florianópolis, Brazil; ^2^Laboratorio Cajal de Circuitos Corticales, Centro de Tecnología Biomédica, Universidad Politécnica de Madrid, Madrid, Spain; ^3^Instituto Cajal, Consejo Superior de Investigaciones Científicas (CSIC), Madrid, Spain

**Keywords:** cognitive and emotional relationships, narrative image, artistic expression, German Romanticism, renaissance art

## Abstract

Knowledge about empathy is part of the study of artistic expressions, among which stand out works of personalities such as the Renaissance polymath Leonardo da Vinci, who was concerned with the connection between science and art during his creative research full of imagination and sensitivity to nature and human anatomy. The word empathy emerged among critics of German art as the term *einfühlung*, which was used within the aesthetic bias by philosophers and art historians. It emphasized the idea that a viewer perceiving an object could establish a link between it and themselves, projecting the object ‘into themselves’. That is, the artwork could be experienced by the observer as if the viewer belonged predominantly to the object, in such a way that its characteristics could be actually felt through the expression of emotions, feelings and thoughts. This analysis of art appreciation required a great deal of knowledge and contemplation of nature, as understood by the German Romanticists, who had enormous admiration for da Vinci and his universal and systematic mind—a mind which reacted against formalisms, building his intellectual and sensory systems based on both his observation of nature and his own criteria. In particular, the art of painting for Leonardo was a way to demonstrate a mental discourse, just as the most important aspect of human portraits is to represent—in gestures and facial expressions—the states of mind and emotions. These are facts that German Romanticists tried to explain as the relationship between empathy and a work of art. The present manuscript aims to describe empathy from an artistic view, considering the roots of this word in German Romanticism; to comment about Leonardo da Vinci and the expression of art in the Renaissance; and, finally, to discuss the expression of his art in relation to empathy.

“Whoever flatters himself that he can retain in his memory all the effects of Nature, is deceived, for our memory is not so capacious; therefore, consult Nature for everything” - Miscellaneous observations, That a Man ought not to trust to himself, but ought to consult Nature, c. 365 (Da Vinci, 2014, p. 208).

## Introduction

Leonardo da Vinci (15 April 1452–2 May 1519) was born in Anchiano, a village near the small town of Vinci, in the period of the Italian Renaissance, in a dynamic society during a time of great intellectual awakening, transformation and resurgence of life and arts and definitive rupture of the medieval conception of the world (McMurrich, 1930, p. 3; Clark, 1976, p. 18). Leonardo is one of the greatest geniuses and polymaths to have ever existed. His work, which is shrouded in mystery, has been the subject of numerous studies in various fields of science, art, and the humanities, and has had an important impact on each of these fields.

**Figure 1:**
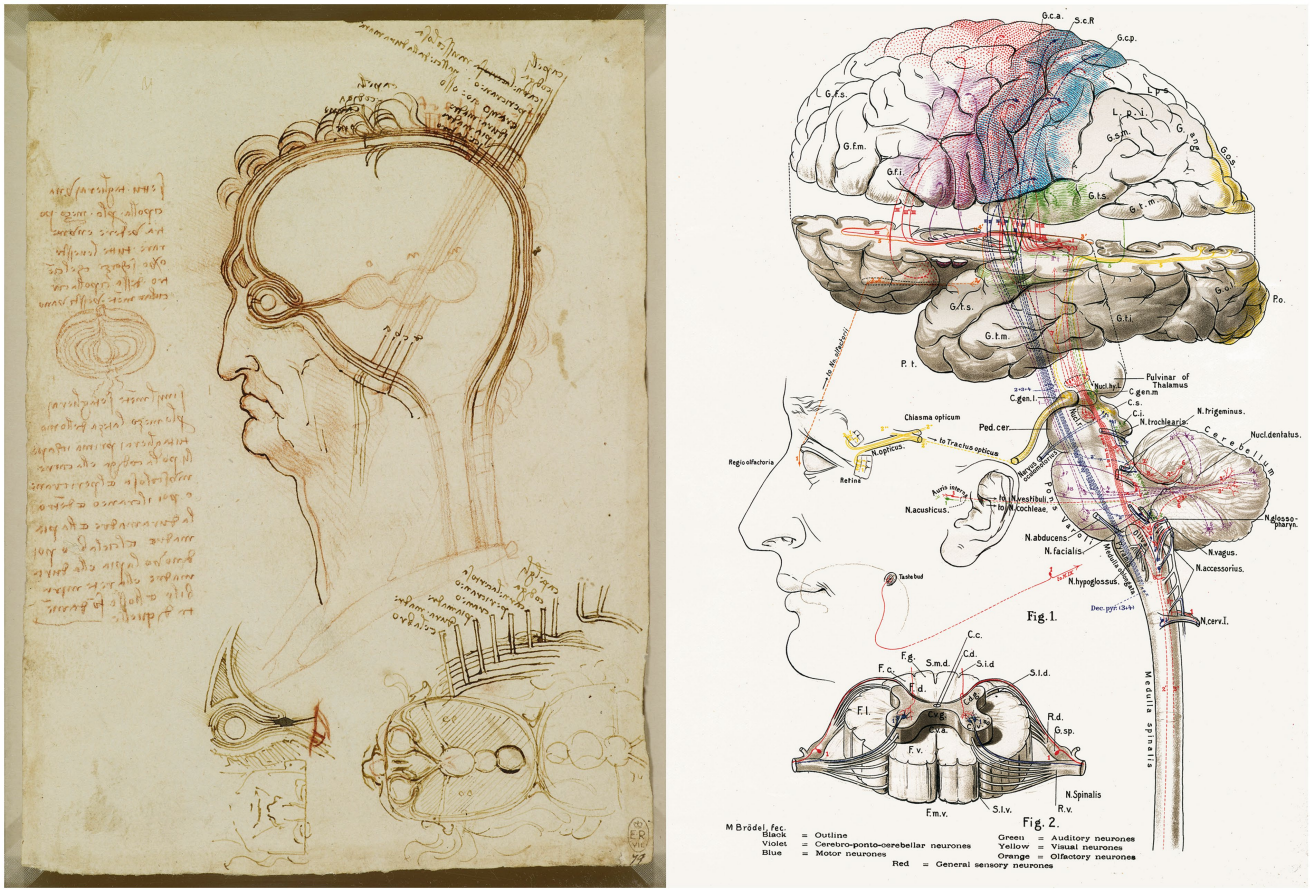
**(Left)** Leonardo da Vinci, *Quaderni d’anatomia* (1490): the central nervous system and cranial nerves. The main drawing shows the layers covering the brain compared to the layers of an onion cut in half (on the left of the image). At the bottom of the drawing, the ventricles viewed from above are illustrated, including the optic and auditory nerves entering the anterior ventricle. Royal Collection Trust/© His Majesty King Charles III, 2023. **(Right)** Barker (1899): schematic drawing to illustrate some of the multiple relationships between different parts of the human central nervous system. Taken from DeFelipe (2017).

In neuroscience, da Vinci was one of the pioneers in trying to explain in detail by physical laws how the brain processes visual information and other sensory stimuli and integrates this information “through the soul.” Da Vinci believed that the anatomical visual system played an essential role in artistic perception and developed a mechanistic model based on the ventricular or cellular doctrine of brain functions (e.g., Pevsner, 2002; DeFelipe, 2017). Shown on the left of Figure 1 is the famous drawing by da Vinci of the central nervous system and cranial nerves, which were believed to be hollow and capable of transmitting “animal or generating forces.” On the right of this figure, a schematic drawing of the human central nervous system by Lewellys Barker (1867–1943) is shown (Barker, 1899). This is a good example of the influence of the anatomical/functional drawings of da Vinci in the early illustrations of ‘modern’ neurological anatomy.

**Figure 2:**
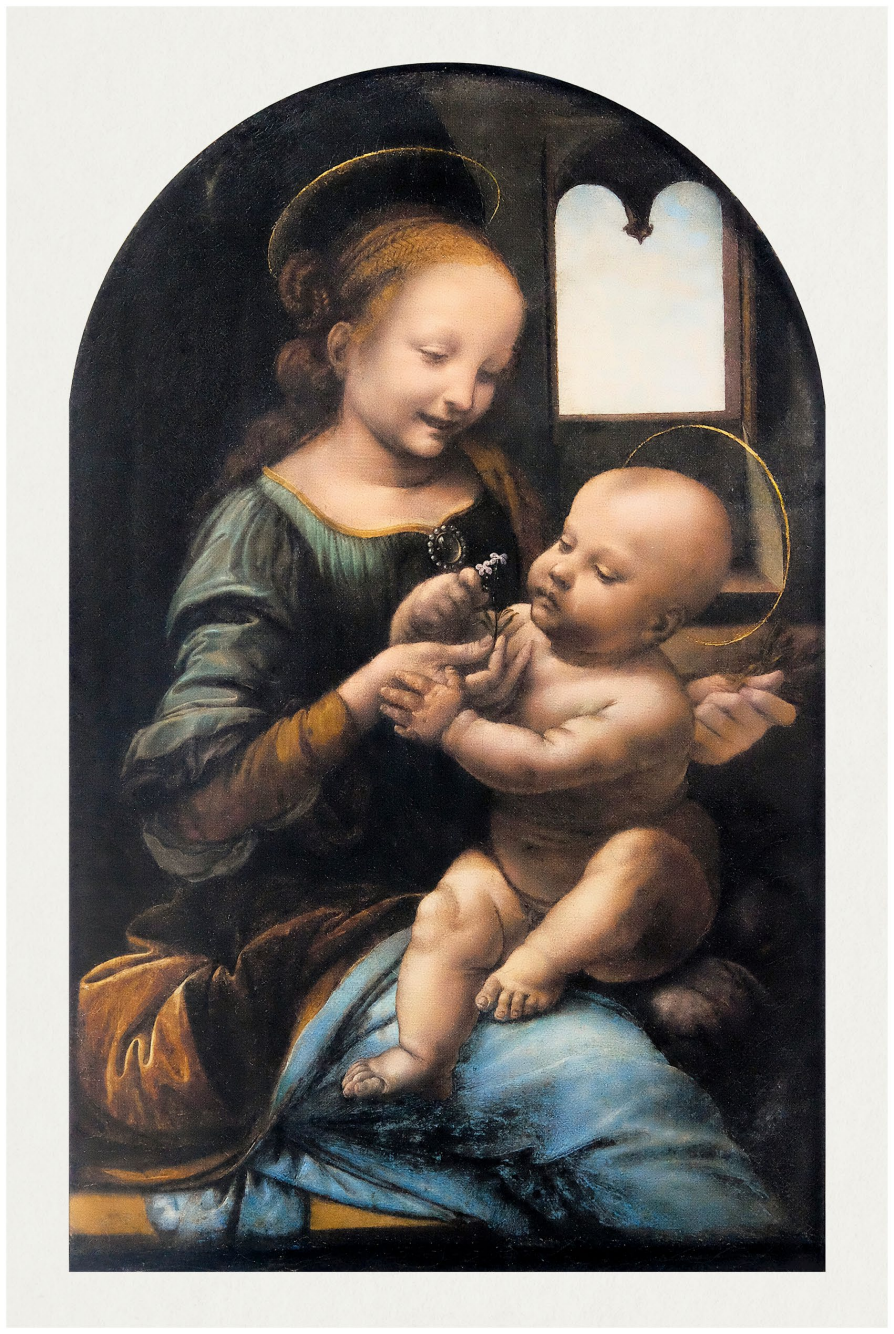
Leonardo da Vinci’s Benois Madonna (circa 1478). Reproduced from rawpixel via Wikimedia Commons, licenced under CC0.

In the present article, we have focused on an extraordinary facet of this genius, namely, the expression of empathy in his artistic works. This is exemplified by the “Madonnas,” which reveal his great ability to illustrate the cognitive and emotional relationships of the characters in his paintings — both between the characters themselves and with the viewer. The first paintings attributed to Leonardo show the use of the traditional disciplines of drawing, perspective and anatomy, as well as a special attention to the effects of light and nature — attention that begins to give rise to the style with which Leonardo renews the Florentine pictorial panorama and manifested in the atmospheric landscapes of works, as in the shadings that give relief to forms, especially intense, in The Virgin with the Child, called Benois Madonna (Sureda, 1998, p. 200; Figure 2). In this regard, da Vinci told his students and disciples (Landrus, 2006, p. 15):

Decide freely on the arrangement of the figures, but always keep in mind that the movements are the expression of the desire of their mind.

The objectives of this manuscript are to explore empathy from an artistic perspective. This exploration begins with a general introduction to empathy and the works of Leonardo da Vinci. A subsequent section will address the word’s roots in German Romanticism, followed by a section dealing with da Vinci and the expression of art during the Renaissance. Finally, a concluding section will discuss the expression of his art in relation to empathy.

## Empathy and Leonardo

Empathy involves experiencing emotion vicariously and understanding the reasons for those emotions (Braadbaart et al., 2014). It may be defined as the capacity to understand other people’s feelings and respond to them appropriately, though it is also considered to be a multifaceted concept that relies on cognition, emotional reactivity and social skills (Baron-Cohen and Wheelwright, 2004; Decety, 2011), while also being based on both perception and understanding (Muncer and Ling, 2006).

As highly social species, we attempt to coordinate our actions and assure successful communication by using language skills and social abilities such as empathy to infer another person’s emotions and mental state (Singer and Klimecki, 2014). Among his many skills and sensibilities, Leonardo da Vinci had the ability to find —through visual language— an important way to communicate his human perceptions and expressions and represent them through art (Vecce, 2003, p. 61; Ruiz García, 2011, p. 225) in which he transmitted figuratively his mental construction about the deep observation of thoughts and emotions, translated by the intention of the mind (Bongioanni, 1978, p. 184; Kemp and Wallace, 2000, p. 16).

Empathy may be served partly by a motor simulation function, and therefore share a neural basis with imitation, as both involve sensorimotor representations of intentions based on perceptions of others’ actions, as well as imagination, after which intentionality and action planning evolve (Braadbaart et al., 2014). One of the characteristics of Leonardo’s way of thinking was based on analogies which were demonstrated in his figures and expressed not only a complement of a text, but an instrument that can stand alone due to its capacity to express rational thought and feelings (Cortes, 1994, p. 37; Ramón, 2011, p. 69). That is, the picture may share an empathic attitude (Titchener, 1909, p. 185) — and, within Leonardo’s drawings, we always find his profound imagination and creativity linked to his systematic observation of nature (Kemp, 2003, p. 147; Capra, 2011, p. 63; Ruiz García, 2011, p. 216).

It is important to note that one of the clearly connected processes in individual brains to phenomena of interest to the humanities relies on the development of studies that potentially dissociate two systems for empathy in the brain, namely, emotional (“I feel what you feel”) and cognitive (“I understand what you feel”), to examine how different empathic responses may be generated, for example, by art (Carew and Ramaswami, 2020). With regard to the drawings of Leonardo, his aim was to clearly consider the relationship between the artist’s expression and the viewer of the artwork (Vecce, 2003, p. 69); for instance, he mentioned that the attitudes and all the posture in a painting ought to correspond with the sentiment expressed in the faces (Da Vinci, 2014, p. 86).

Leonardo was a pioneer in demonstrating what was possible with anatomical illustrations and his approach inspired other artists to encourage the viewer to become a witness of what he saw (Kemp and Wallace, 2000, p. 33). In this regard, he affirmed that the painted figures should be created so that the observer could easily know—through the movements and attitude of the figures—the mental situation of the creator of the narrative and the meaning of his intentions (Da Vinci, 2013, p. 121), since an experiencer must empathize with an observer in order to think, understand and communicate as he does (Titchener, 1909, p. 185).

Most of the themes presented in this introduction are expanded upon in the last chapter of this manuscript, entitled Leonardo, empathy and the expression of his art. The following sections include an essay on empathy from an artistic perspective, considering the roots of this word in German Romanticism, followed by a commentary on da Vinci and the expression of art in the Renaissance — and, finally, a discussion of the expression of his art in relation to empathy.

## Empathy and German romanticism

“One painter ought never to imitate the manner of any other; because in that case he cannot be called the child of Nature, but the grandchild. It is always best to have recourse to Nature, which is replete with such abundance of objects, than to the productions of other masters, who learnt everything from her” - Miscellaneous observations, Painters are not to imitate one another, c. 354 (Da Vinci, 2014, p. 203).

The word empathy has its roots in the Greek ‘*empatheia*’ (‘*en*’, in; and ‘*pathos*’, feeling). This term was introduced in the aesthetics of German art from the translation of the word *einfühlung*, or “feeling into,” to English by the Anglo-American Edward Titchener (1867–1927), in 1909 (Stueber, 2013). The term *einfühlung* was used within an aesthetic context by the philosopher Rudolf Lotze (1817–1881) in 1858, who discovered the projection of our inner experience into forms, leading to us sharing their essence (Lee and Anstructer-Thomson, 1912, p. 17). Similarly, in 1873, the term was also used by the art historian and philosopher Robert Vischer (1847–1933), whose ideas —which were widely accepted by art historians— included the projection of himself into the artistic object (Preston and De Wall, 2002, p. 1). In his words, “I can think my way into [an object], mediate its size with my own, stretch and expand, bend and confine myself to it” (Vischer, 1873, p. 104). By *einfühlung*, Vischer meant the physical responses that are generated by the observation of paintings and he described how particular forms aroused particular responsive feelings, depending on their conformity to the design and function of the muscles of the body (Freedberg and Gallese, 2017, p. 198).

Developing Vischer’s ideas, the art historian Heinrich Wölfflin (1864–1945), in 1886, set out his views on how observation of specific architectural forms engages the beholders’ bodily responses, while Karl Gross (1861–1946), in 1892, used Vischer’s understanding to represent aesthetic satisfaction as an activity of inner imitation (Freedberg and Gallese, 2017, p. 198). Wölfflin agreed that we subject all objects to “soulification” in this projective way, and suggested that such projection involved actual workings of the motor nerve system (Peacocke, 2023). Around the same time, Bernard Berenson (1865–1959) outlined his views on how observation of the movements shown in Renaissance works of art enhanced the beholders’ sense of the capacities of the comparable muscles within their bodies (1909, p. 25). However, there were similar descriptions previously; for example, in 1774 the philosopher and novelist Johann Herder (1744–1803) associated the word in German with enlightenment to understand other times and cultures (Zölzer and Zölzer, 2020, p. 2).

Empathy among critics of German Romantic art is taken to entail aesthetic taste being an objectification of one’s own personal taste, and in particular, taste for a work of art is the contemplation of an object, consisting of perceiving the vitality of this object, as the symbol of a life that is there (Lipps, 1924, p. 96). From this perspective, critical analysis of art among romanticists implies having knowledge of the object on which the work is based, with knowledge of nature considered essential (Lipps, 1924, p. 13). In turn, the object becomes a potential source of free thinking, preceded by spontaneity and knowledge, in such a way that it translates a reflection of oneself (Benjamin, 2017, p. 66) and an interpretation of the external world that mirrors the characteristic of each individual mind (DeFelipe, 2014, p. 67). Da Vinci expands on this notion as follows (from Linear Perspectives, in Of Mental Motions, c. 110; Da Vinci, 2014, p. 48):

“A mere thought, or operation of the mind, excites only simple and easy motions of the body; not this way, and that way, because its object is in the mind, which does not affect the senses when it is collected within itself.”

However, between 1903 and 1906, psychologists attributed to the German Theodor Lipps (1851–1914) the discovery of empathy, related to the German word *einfühlung*, since it was he who organized and founded the term for the area of psychology (Stueber, 2013). Lipps first proposed that empathy described the relationship between a work of art and its observer, but soon expanded this concept to encompass interactions between people by interpreting that our perception of the movements of others is a form of inner imitation (Iacoboni, 2009, p. 111). He attributed our capacity for empathy to a sensory-motor mirroring — an involuntary, kinesthetic inner imitation of the observed that informs our experience of art (Stamatopoulou, 2018, p. 170). Empathy theorists took it that aesthetic experience involved mentally projecting ourselves into the physical shape of an item to have an emotional or dynamic experience of the kind that a human subject would have if taking on that physical shape (Vischer, 1873, p. 104).

Another German, Antonin Prandtl (1880–1927), less well known than Lipps, explained —in 1910— that even though people can only know their own inner life, what is only known is the very image or thought, while understanding of the other’s life can occur through empirical empathy or via empathy through feeling (Cortes, 1994, p. 30). Empirical empathy depends on a previous occurrence of feeling and assumes that it has already been felt by the person himself; empathy through feeling is closer to the version expounded by Lipps. In both types of empathy, there is the characteristic that what occurs in the viewer is driven by the object, whether it is another person or a work of art (Cortes, 1994, p. 30).

Lipps and Prandtl, among others, used the term empathy to explain how a person grasped the meaning of aesthetic objects and the consciousness of other people. Titchener, employing the term empathy, thought that one could not know the consciousness of another person trying to enter their mind through reason, but through inner imitation or motor mimicry, with an effort of the mind (1909, p. 185), which most people did to detect their ‘core’ of empathy. He adapted William James’s (1842–1910) notion of ideomotor action—through which mental representations are scaffolded by embodied percepts—to claim that kinesthetic imagery supports empathy (Stamatopoulou, 2018, p. 170). In fact, through imitation and mimicry, we can feel what other people feel and also understand their emotional states (Iacoboni, 2009, p. 116). In this regard, Titchener wrote (Titchener, 1909, p. 21):

“… the various visual images, which I have referred to as possible vehicles of logical meaning, oftentimes share their task with kinesthesis. Not only do I see gravity and modesty and pride and courtesy and stateliness, but I feel or act them in the mind’s muscles.”

It is relevant to mention that, in 1895, the British writer Vernon Lee (1856–1935), pseudonym of Violet Paget, translated the German word to sympathy, explaining that there is a vivification of feelings from what we perceive which, when transformed into our own muscular efforts, allows us to fully feel, indicating *einfühlung’s* relationship with muscular mimicry (Lee and Anstruther-Thomson, 1912, p. 107). Sympathy was the term commonly used to refer to empathy- related phenomena before the introduction of the term empathy into the English language as the translation of *einfühlung*, reflecting the fact that in encountering other persons, humans can resonate with and recreate that person’s thoughts and emotions on different dimensions of cognitive complexity (Stueber, 2013). In this regard, Edgar Allan Poe (1809–1849), in his famous short story The Purloined Letter (Poe, 2021, p. 140), wrote:

“When I wish to find out how wise, or how stupid, or how good, or how wicked is any one, or what are his thoughts at the moment, I fashion the expression of my face, as accurately as possible, in accordance with the expression of his, and then wait to see what thoughts or sentiments arise in my mind or heart, as if to match or correspond with the expression.”

Poe could not have chosen a better way to penetrate the life of his characters. However, he was not the only one; the literature on emotions reveals an awareness that emotional experience takes shape through expressions of the facial muscles (Iacoboni, 2009, p. 121). For example, Nicolas Malebranche (1638–1715) already understood that “a passion in one individual will produce facial expressions and other sensible bodily effects, such as changes of color, words, cries, and the like” (1997) and, for Charles Darwin (1809–1882), “the free expression by outward signs of an emotion intensifies it; on the other hand, the repression, as far as this is possible, of all outward signs softens our emotions” (Darwin, 1872, p. 366). It is also evident that the brain can internally simulate certain emotional bodily states, as occurs in the process of transforming the emotion of sympathy into a feeling of empathy (Damasio, 2003, p. 174). In line with these observations, Titchener described becoming keenly alive to the variety of organic attitude and its kinesthetic representation (Titchener, 1909, p. 180).

The notion that feedback from emotion-specific facial reactions can causally influence other components of emotional responses, such as subjective experience and autonomic reactions, has been supported by authors such as Tomkins (Laird, 1984). Additionally, the concept of emotional facial displays serving as input to the experience of emotion has been postulated (Hess et al., 1992). The face has long been recognized as a crucial source of information for observers about a person’s underlying emotional state (Hess et al., 1992). This is because the perception of a facial expression leads to unconscious emotion-specific facial mimicry and contributes to a representation in the neural production system for facial expressions (Krauthein et al., 2020).

Centuries before, Leonardo already understood that if a painter had to characterize the intentions of the mind in a narrative image, it was necessary to profoundly understand the causes that would generate such external effects; that is, to recreate the bodies and faces of the protagonists, one should follow the sensations and emotions of the dramatized situation, as he represented in The Last Supper (Kemp and Wallace, 2000, p. 70; Nadal, 2014, p. 142). In this regard, he understood that the body was the first work of the soul, its external and visible expression—modeled by the spirit of the body itself (Müntz, 2005, p. 268; Capra, 2011, p. 33).

It is interesting to note that, regarding empathy, Lipps comments that observers project themselves onto the object of their perception, that is, certain characteristics are experienced by the observer as if he or she belonged to the object, so that the object was actually felt (1924, p.1). Moreover, for Lipps, *einfühlung* means that the experience of a viewer of a gesture of pride or a joyful smile is similar to that of the individual who is experiencing the emotions related to their gestures, providing the perception of the other (1924) and is thus in agreement with Leonardo’s idea that all our knowledge originates in our perceptions and feelings (Ruiz García, 2011, p. 228).

After 1,505, the expression of emotions was studied in depth by Leonardo—under the designation of “*moti mentali*” (motions of the mind)—and pointed out in several chapters of the Treatise on Painting, with the variety of faces showing the human emotional states accompanied by the attitudes of the pictorial characters and their entire body, which corresponded to the feeling expressed in their portrayed faces (Richter, 1977, p. 344). Similarly, for the romantics, art is a means to arouse emotions (Clark, 1973, p. 24) and the totality of a work of art is where all of its infinite meanings can be found — or, as Goethe defines it, it is in the plurality of a work of art that its unity is found (Benjamin, 2017, p. 150).

Although each country studied the magnificent material left to the world by Leonardo, the Romantic Movement in Germany brought an entirely new and essential idea to its study, which would become part of German artistic heritage (Adriani, 1978, p. 200). As it developed, Romanticism proposed a series of innovations with regard to human values, forming a rebellion against the static conformity of the 18th century and, in the artistic field, a rebellion against the prevailing forms imitated from Greek sculpture and the prohibition of colors and movement as expressions of life force (Clark, 1973, p. 24). Leonardo’s descriptions not only reveal the deeply romantic side of his imagination, but also imply a sense of form completely at variance with that of his contemporaries, since instead of the firmly defined forms of the *quattrocento* or the enclosed forms of the High Renaissance, the subjects he describes could only be treated with the broken, suggestive forms of romantic painting (Clark, 1976, p. 81). He advised the painter to study not only marks on walls, but also the embers of the fire, or clouds, or mud, or other similar objects from which you will find most admirable ideas because, for him, from a confusion of shapes the spirit is quickened to new inventions (Clark, 1976, p. 82). It is important to note that nothing could be farther from the precepts of academic classicism than the use of stains on walls to stimulate the imagination—a procedure employed by Goya (1746–1828), one of the most anti-classical of all painters; and Victor Hugo (1802–1885), whose name is the first that comes to mind when we read Leonardo’s descriptions of a deluge (Clark, 1976, p. 82).

Leonardo’s greatness in the spiritual history of Germany has been inexhaustibly verified, as noted in the writings of the German Joachim von Sandrart (1606–1,688), one of the founders of art history in northern Europe, whose perspective closely resembled that of Leonardo given his view of painting as a science based on observation and his concern for organization and simplification in teaching with regard to the representation of the human body, light, and landscapes (Heck, 2009, p. 378). Sandrart’s idea of the ideal painter comes from the Treatise of Leonardo and corroborates the fact that the painting of nature, performed with reason and meticulous observation, is the best method for a painter to learn their craft (Heck, 2009, p. 384). Accordingly, for Leonardo, painting is a re-creation of the visible world with the painter’s imagination. It is this view of art as a creation which makes him insist that the painter must be universal, must neglect no aspect of nature and—for the same reason—he must be a scientist, that is to say, he must understand the inner nature of what he paints almost as if he had created it himself (Clark, 1976, p. 75).

For the romantics, the idea of art is the idea of its form, just as the ideal of art is the ideal of its content—pure distinctions of the philosophy of art, however, which have been little understood since the German artistic philosophy of 1800 (Benjamin, 2017, p. 152). Even Goethe did not succeed in clarifying this question—and neither did the romantics, since only a person with systematic thinking would be able to conceive the ideal of art (Benjamin, 2017, p. 152). So, naturally, an artist like da Vinci greatly appealed to the romantics (Adriani, 1978, p. 200), who aspired to infinity and loved the enigmatic, since Leonardo was rigorous and systematic, which made him react against formalisms and build his intellectual and sensory systems based on both direct observation of nature and his own criterion (Pérez de Guzmán, 2003a, p. 18).

Goethe, a fundamental contributor to German Romanticism, thought that it is measure that underlies beauty, which has its manifestation through content; however, the concept of measure is far from Romanticism, which did not accept that anything should be measured in art (Benjamin, 2017, p. 153). In this regard, it is interesting to note that for Leonardo the sense of proportion in art refers not only to numbers and measurements, but also to sounds, weights, moments, positions, humor and any other elements that may emanate the power and beauty of an image (McMurrich, 1930, p. 109; Richter, 1977, p. 228), as diverse and limitless as imagination (DeFelipe, 2014, p. 72). Like anyone who has attained a high degree of wisdom, Leonardo learned that the supreme sign of sublimity in a creation is harmony — the end toward which he aimed, as his supreme goal, in using lines and concepts, in molding the figurative expression to the spirit of the work (Biaggi, 1978, p. 447).

**Figure 3:**
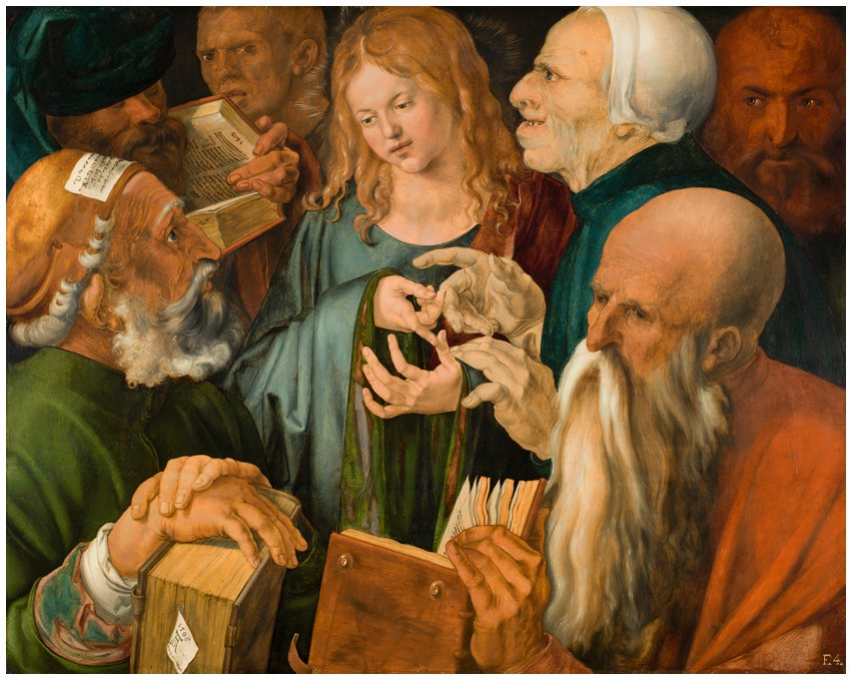
Albrecht Dürer: Christ among the Doctors (1506). Reproduced with permission from Museo Nacional Thyssen-Bornemisza. Copyright © Museo Nacional Thyssen-Bornemisza, Madrid. Source: https://www.museothyssen.org/en/collection/artists/durer-albrecht/jesus-among-doctors.

Goethe in particular found his own aspirations in Leonardo, with whom he had deep spiritual and artistic affinity, as noted by his admiration for the Last Supper, in particular the unusual configuration of the individuals’ faces, revealing their character (Adriani, 1978, pp. 195, 200). Other well-known figures were also influenced and impressed by Leonardo’s study of faces, especially due to this masterpiece which expresses intense feelings and beauty. One such figure was Albrecht Dürer (1471–1,528) —who has the most extensive and complex bibliography among German artists— in his own Gothic version of the work Christ among the Doctors (Figure 3), in which the profile of the doctor to the right of Jesus is clearly reminiscent of the work of Leonardo (Legaldano, 1972, p. 82; Clark, 1976, p. 119; Adriani, 1978, p. 200). The compositional idea of Dürer is undoubtedly Italian considering the tight group of half-figures, which resemble the style of Leonardo, but the translation is fundamentally and musically Gothic, with the fluctuating figures in the speculum almost without perspective, arranged around the four central hands — very unusual indeed in a Renaissance scheme (Legaldano, 1972, p. 115).

Among other representatives of German Romanticism, Caspar David Friedrich (1774–1840) reported that a painting must not be invented but felt; every manifestation of nature, recorded with precision, with dignity and with feeling can become the subject of art (Grummt, 2009, p. 10, 150). He also maintained that all authentic art has an inner impulse to create it, often without the artist being aware of it (Busch, 2009, p. 30). His work as a draftsman further distinguishes him within this rich, flourishing genre in Germany around 1800 (Börsh-Supan, 2009, p. 19), although, with all his singularity, he seems to approximate to the interests of Leonardo, such as in the Study of a Woman Reading and Study of a Cow and a Horse’s Head (see catalog of the exhibition “Caspar David Friedrich. The Art of Drawing” Madrid: Fundación Juan March, 2009, p. 56), which includes nature, the study of a human and animals. Also for Georg Philipp Friedrich von Hardenberg, known by his pseudonym Novalis (1772–1801), the process of observation is both a subjective and objective process, an ideal and real experiment, that passes from knowledge of nature to spiritual knowledge, at which point a work of art can be contemplated (Benjamin, 2017, p. 75). Interestingly, Novalis proposed understanding empathy from *feeling* nature, as a correction to scientistic attitudes (Stueber, 2013).

These thoughts are in agreement with Leonardo since they suggest who stated that each instrument in itself must function according to the experience from which it originates and therefore advocated that when drawing a figure, one should think carefully about what it is and what one wants it to represent and, at the end, verify that this figure conforms according to the intention and the claim of its creator (Richter, 1977, pp. 239, 351). Leonardo’s concern as a draftsman was to give priority to the study of gestures, attitudes and actions so that the figures could better convey the thoughts and emotions that provoked them (Pedretti, 2003, p. 95); furthermore, his conception of art as a science made him add a warning that the painter must understand the detailed structure of all that he wished to represent (Clark, 1976, p. 82).

## Leonardo and the expression of art in the renaissance

“The painter ought always to form in his mind a kind of system of reasoning or discussion within himself on any remarkable object before him. He should stop, take notes, and form some rule upon it; considering the place, the circumstances, the lights and shadows” - Invention or Composition, How a Painter ought to proceed in his studies, c. 130 (Da Vinci, 2014, p. 61).

“A painter should delight in introducing great variety into his compositions, avoiding repetition, that by this fertility of invention he may attract and charm the eye of the beholder” - Invention or Composition, Of Variety in History, c. 137 (Da Vinci, 2014, p. 63).

Although it is difficult to specify precise historical dates, many Italians began to change their attitude toward the world in the late 13th century and especially in the 14th and 15th centuries (Mannering, 1981, p. 10). The most notable artists and authors of the first phase of the Renaissance movement, the so-called *quattrocento*, with spiritual freedom and free of medieval superstitions, created humanism, which allowed them to return to their reality and create their own world (Pérez de Guzmán, 2003b, p. 21), look around and rediscover nature, spiritual mystery, the harmony of ideas, and the beauty of architecture, sculptures and painting (Franqui, 2003, p. 19).

**Figure 4:**
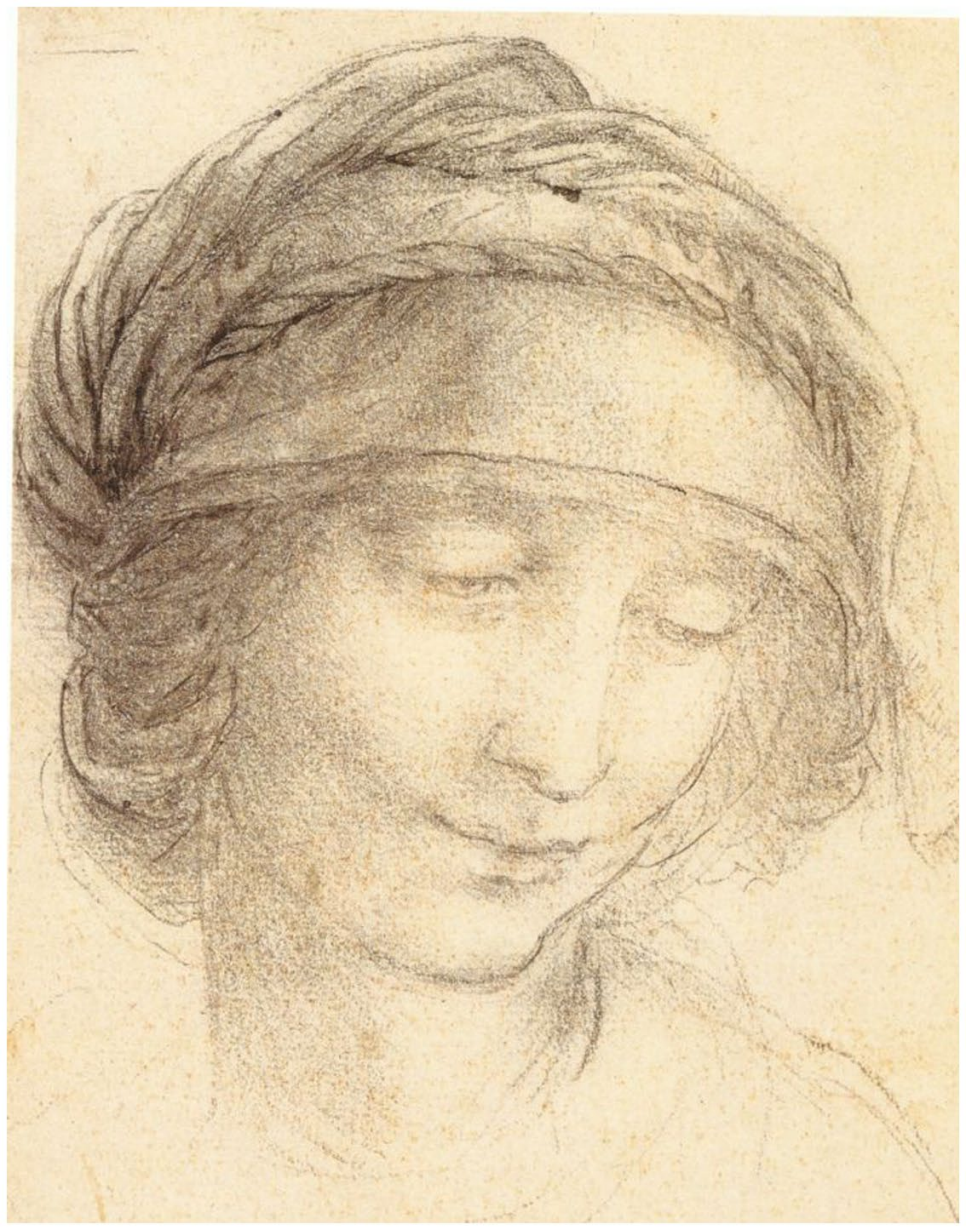
Leonardo da Vinci, The head of Saint Anne c.1510–15. Black chalk | 18.8 × 13.0 cm (sheet of paper). Public domain, via Wikimedia Commons.

If in Greece the new culture was born among poets and philosophers, the Renaissance revolution, which emerged in Florence and then spread to other cities in Italy and Europe, had architects, sculptors and painters as protagonists, like Brunelleschi, Donatello, Massacio, Ucello, Piero della Francesca, Boticelli, Michelangelo, Rafael and Leonardo da Vinci (Franqui, 2003, p. 19). For Florentine artists, the basis of creativity and the central element for the quality of their training —and even their identity— was drawing, which was intensely admired, with well-regarded examples including the drawings of The head of Saint Anne (Figure 4) and the Battle of Anghiari, by Leonardo (Franklin, 2005, p. 18). With the Renaissance, artists felt the need to find new ways to express their thoughts and feelings and were driven to study nature and truth more deeply, since these resources could supply the artist with more effective representations than those obtained from repetitions or copies that could easily become conventional (Pedretti, 1964, p. 115; Biaggi, 1978, p. 437). The introduction of an original and vivid beauty in painting showed that the figures that once pleased artists and the public began to lose their appeal, while interest in the idea of invention or originality as an aesthetic criterion grew (Waldman, 2005, p. 32). Leonardo, whose independent mind always studied, absorbed and recreated, never copying or imitating (Steinitz, 1960, p. 116), holds a place in history against all formalism in general (Bongioanni, 1978, p. 185).

When invention or originality became a condition of privilege for artists, art came to be characterized as an intellectual pursuit and not a production of work and, as a result, the status of the artist was elevated (Waldman, 2005, p. 32). The best representative of this new conception of art as an intellectual activity was Leonardo da Vinci, who defined painting as a process of investigation of the natural world, representative of his science (Kemp, 2003, p. 147; Waldman, 2005, p. 32). Leonardo describes painting as a subtle invention which —with philosophy and speculation—considers nature in all its forms (Kemp, 2003, p. 147). Indeed, his idea of imitating nature fits into the idea of invention, which is more than merely knowing, since we know by means of the intellect, but invent by means not of the intellect but of reason, and nature is not intellect, it is reason (Bongioanni, 1978, p. 185).

This new behavior exerted great influence on the visual arts and by the end of the 13th century, many artists changed the direction of their art and began to become increasingly interested in physical reality and the accurate reproduction of aspects of things, giving room to subtleties of the shape and size of objects; consequently, the study of perspective and anatomy acquired fundamental importance among artists of the Florentine Renaissance (Richter, 1977, p. 227; Mannering, 1981, p. 12). Moreover, as part of the naturalist revolution, the body was understood as a functional system between movement and emotion that included not only muscular and skeletal mechanisms, but also expressive characteristics of character and feelings, allowing the knowledge of artists and anatomists to be united, as demonstrated by Andreas Vesalius (1514–1,564) in *De Humani Corporis Fabrica*, from 1,543, which was illustrated by the artist Jan Stephan van Calcar (1499–1,546) (Kemp and Wallace, 2000, p. 13).

As a corollary of the physical body being considered as an expression of thoughts and emotions, the recognition of the potential value of figures for the demonstration of everything that can be explained through words has increased (Kemp and Wallace, 2000, p. 13). This corroborates the highest ideal of Leonardo’s existence as a painter, namely the importance of painting as an instrument of knowledge (Richter, 1977, p. 101; Ramón, 2011, p. 24), a form of expression that he valued very highly due to the immediacy of its representation and potential to communicate (Vecce, 2003, p. 59). Leonardo was one of the masters who used the creation of a pictorial universe with perfection to express the truth of nature and a deep spirituality (Mannering, 1981, p. 22); for him, as a genius of acquiring knowledge from observation and experiment, the soul was the main ‘sense’ to appreciate more fully the infinite work of nature (McMurrich, 1930, p. 82; Tarazona, 2003, p. 52). Interestingly, other exponents of the Renaissance, such as Galileo Galilei (1564–1,642), used Leonardo’s arguments to demonstrate the primacy of painting (Heck, 2009, p. 380).

**Figure 5:**
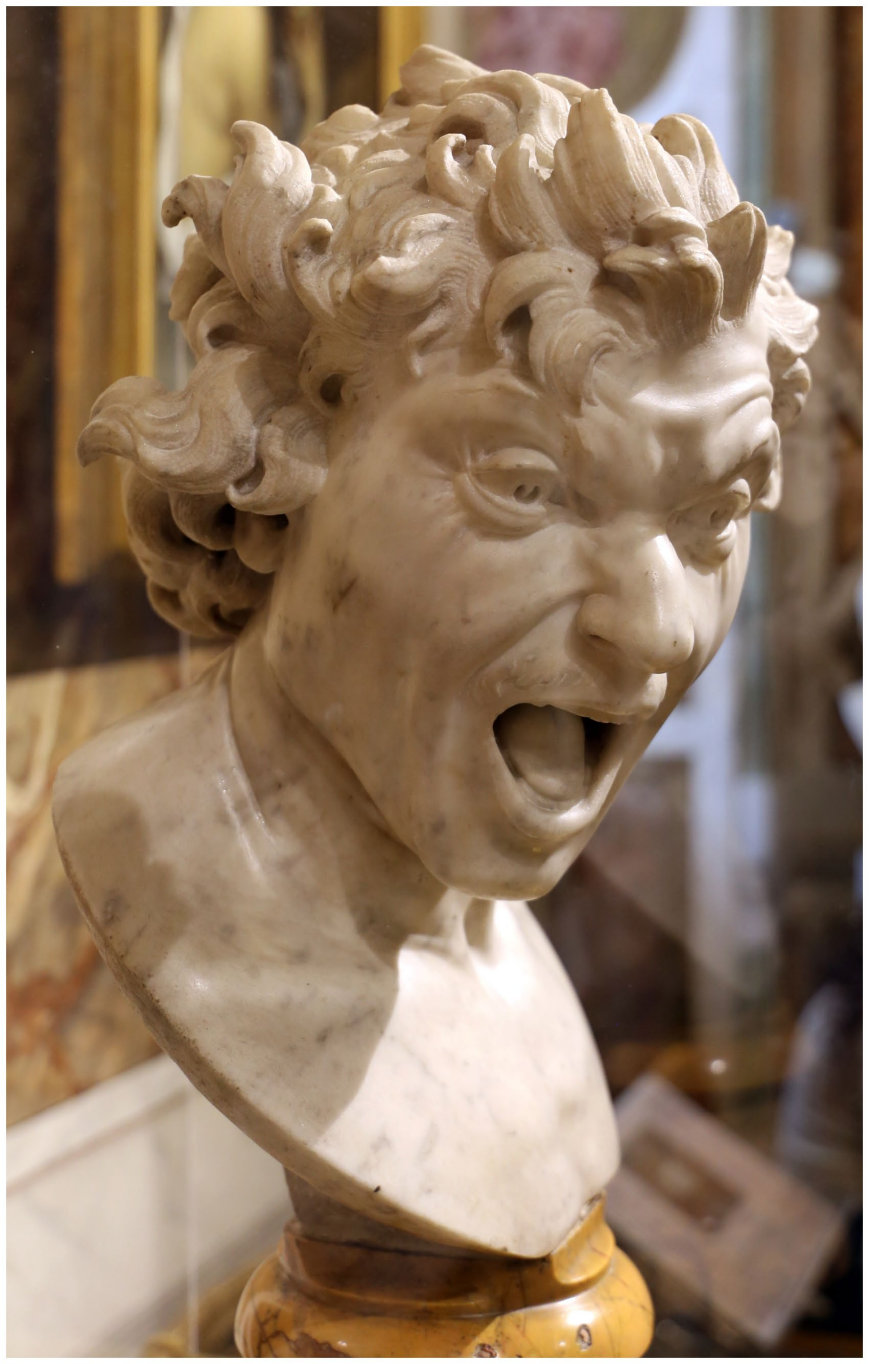
Gian Lorenzo Bernini. *Anima Dannata*, 1,619 *ca.* (Roma, Palazzo di Spagna) by Sailko is licensed under CC BY 3.0. Taken from https://openverse.org/image/58719f8e-cf5a-410d-9ec1-3a47a6ce7623?q=anima%20dannata.

**Figure 6:**
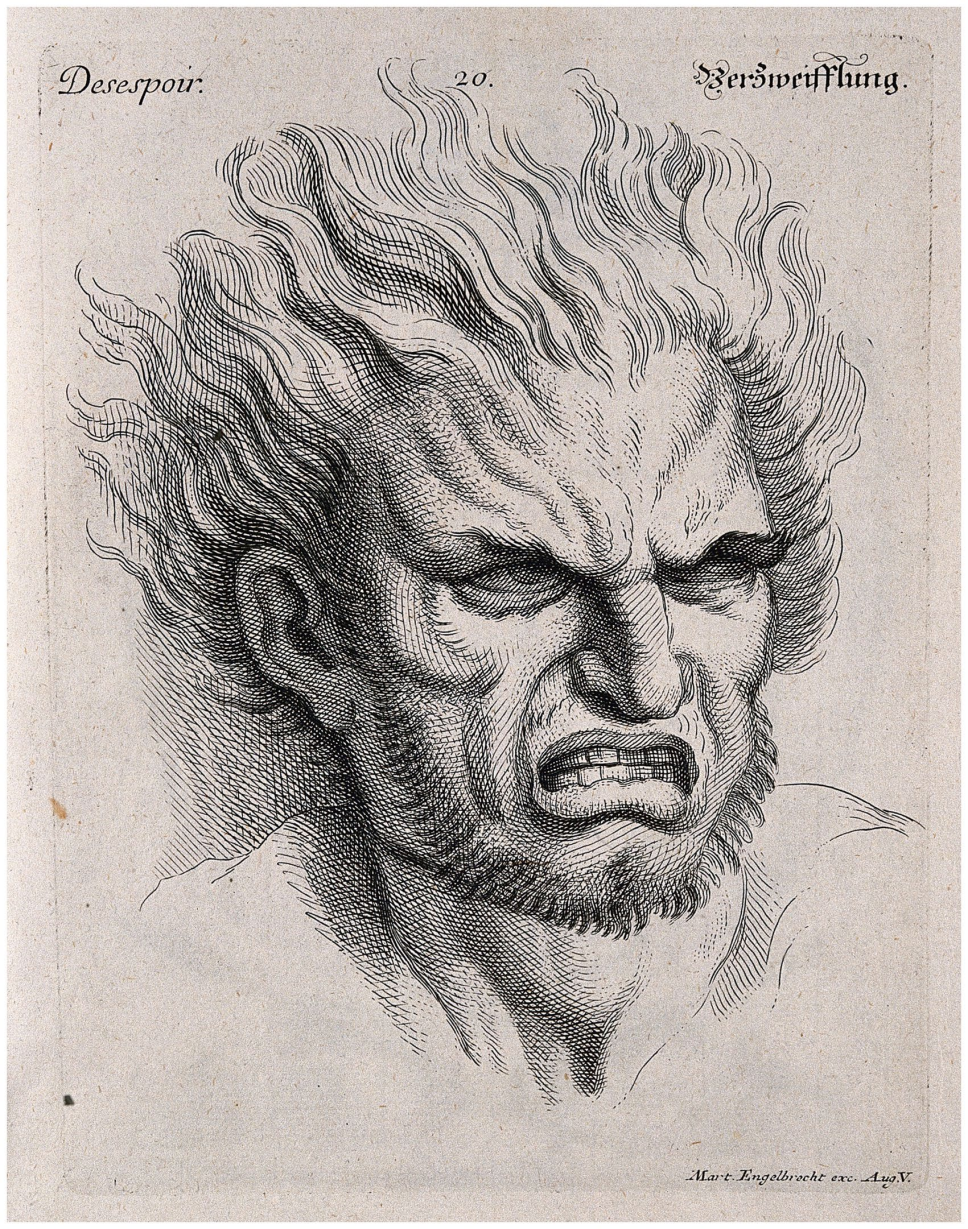
Head of a man with hair raised, expressing despair. Engraving – thought to be by M. Engelbrecht, 1732, after Charles Le Brun. Public Domain Mark. Source: Wellcome Collection.

Among the possibilities of expressing by illustrations, something that fascinated the artists were facial expressions, considered the most eloquent form after the hand (Kemp and Wallace, 2000, p. 97). Baroque artists such as Gian Lorenzo Bernini (1598–1,680) and Rembrandt van Rijn (1606–1,669) intensively studied faces *in extremis* to create narrative compositions with the essence of emotions, as did other artists like Charles Le Brun (1619–1,690) and Wilhelm von Kaulbach (1805–1874) (Kemp and Wallace, 2000, p. 97; Figures 5, 6). It is relevant to note that we are observant beings and instinctively physiognomists, equipped with a visual system that perceives subtle morphological variations of the face, identifying their characteristics and the expression of emotions — from brain regions that functionally can differentiate one faces from one another and discern their specificities (Kemp and Wallace, 2000, p. 15).

Leonardo’s pictorial works were eagerly awaited and brought together a variety of facial expressions reflecting human emotions attitudes and general state of the body, with expression through the limbs and particularly the hands of the subjects (as well as their posture) corresponding to the feelings expressed on their faces, such as fatigue, rest, anger, pain, fight or flight, crying, smiling, fear, command, neglect, among others (Pedretti, 1964, p. 133; Richter, 1977, p. 344). It is important to note that one of the changes introduced by Leonardo and that determined the path of Italian art of the 15th century, especially Florentine art, was an appreciation of the effects of light and shadow, the so-called *chiaroscuro* (Clark, 1976, p. 76; Mannering, 1981, p. 77). The relief, the illusion of three-dimensionality and the highlight of a flat surface together represent the essential quality of a painting and must be the first intention of a painter and this can only be achieved by capturing the play of light on the surfaces so that shadows and variations in tone suggest surface irregularities (Da Vinci, 2013, p. 154). Many of his faces and landscapes were created with imperceptible changes of tone, using a technique called *sfumato* that allowed him to achieve light plays that reflect his belief that in a painting there should be no sharply defined contours (Clark, 1976, p. 77; Mannering, 1981, p. 77). Regarding this, in Light and Shadow, Of the Beauty of Faces (c. 194), Leonardo reports (2014, p. 98):

“You must not mark any muscles with hardness of line, but let the soft light glide upon them, and terminate imperceptibly in delightful shadows: from this will arise grace and beauty to the face.”

**Figure 7:**
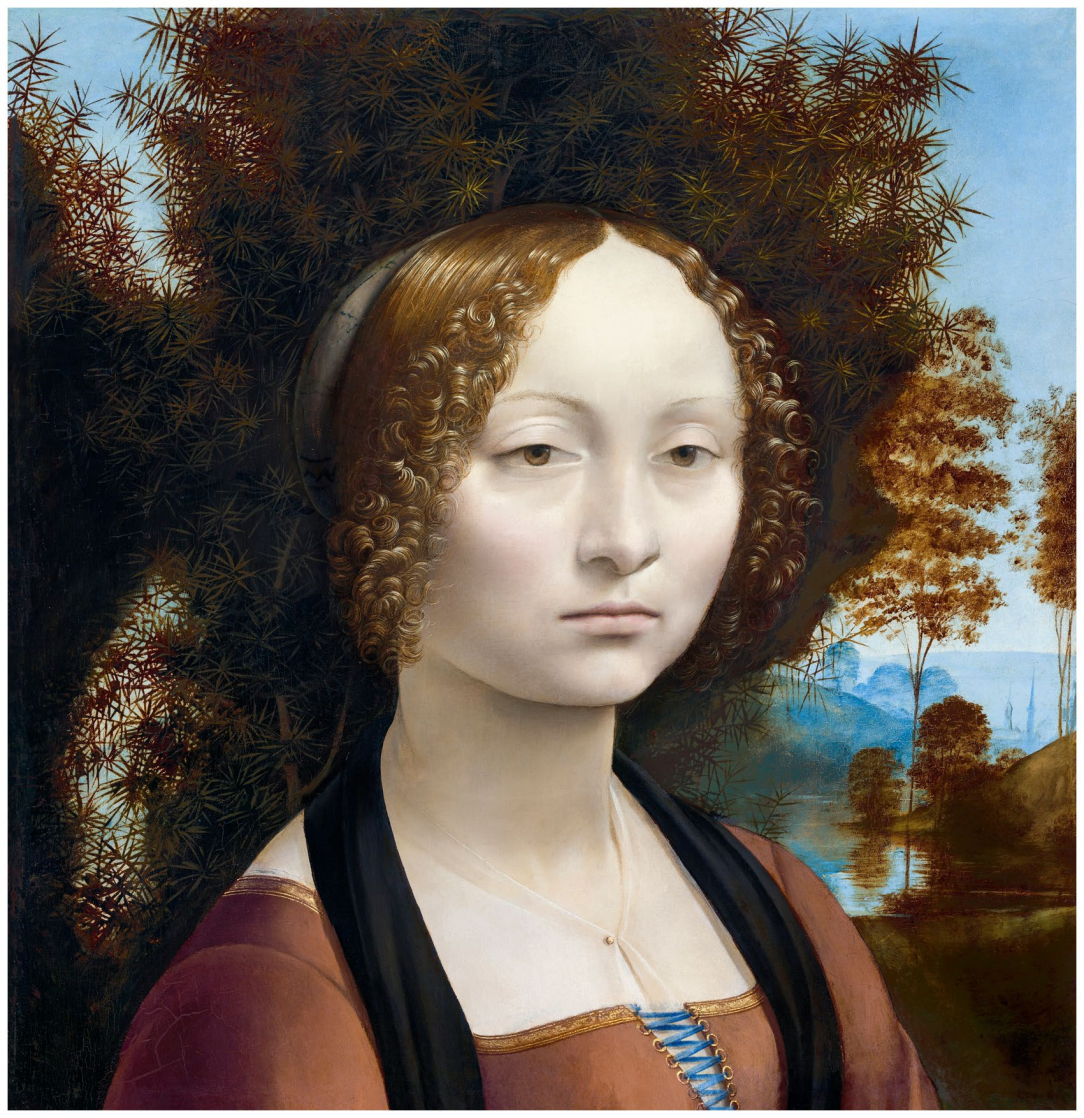
Ginevra de Benci (*ca.*1474–1,478) painting in high resolution by Leonardo da Vinci. Original from The National Gallery of Art. Reproduced from rawpixel via Wikimedia Commons, licenced under CC0.

**Figure 8:**
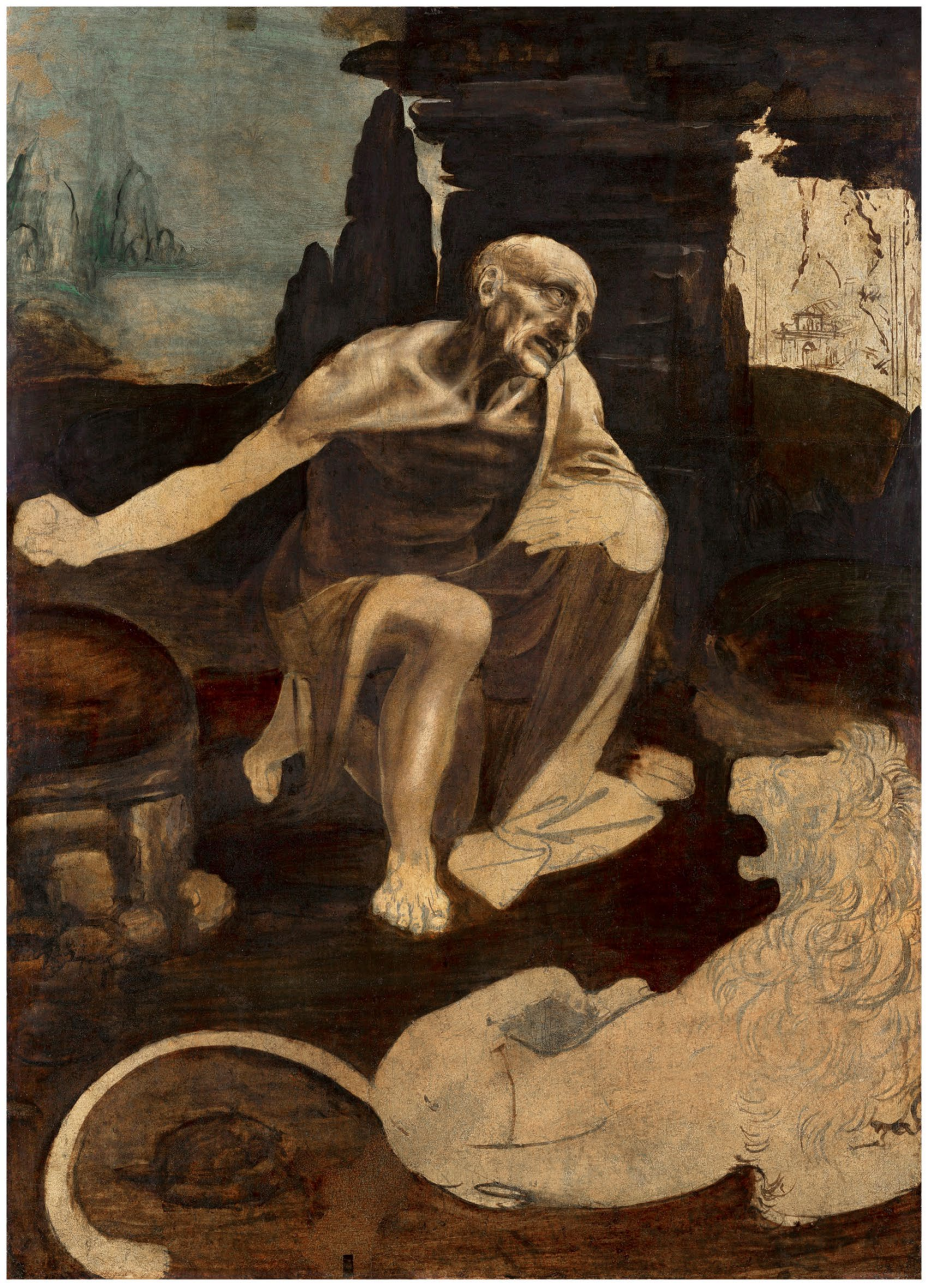
Leonardo da Vinci’s Saint Jerome in the Wilderness (circa 1480). Reproduced from rawpixel via Wikimedia Commons, licenced under CC0.

These new techniques allowed the most reliable expression of the reality of thoughts and emotions in a pictorial universe, as Leonardo demonstrated. For example, Ginevra de Benci (1474) is portrayed with beautiful colors and enigmatic details (Figure 7); in The Adoration of the Magi (1481–1,482) and Saint Jerome (1,483, Figure 8), Leonardo captured a dramatic moment with unparalleled realism and emotional strength (Mannering, 1981, p. 25; Prat et al., 1989, p. 68); in The Last Supper (1495–1,497), which is what Dante Alighieri (1265–1,321) would have called an “alta fantasia,” each character reveals a state of mind, each of which required in-depth psychological study to portray (Kemp, 2003, p. 176), with Leonardo basing his composition on the motive of a central type of innocence and beauty surrounded by embodiments of worldly passions, in this case cunning and obstinacy — such as in the Uffizi Adoration (Clark, 1976, p. 119); and Gioconda/Monalisa (1503–1,505) is a painting with the power of the human soul of Leonardo, his masterpiece revealing his thought and work (Pater, 2013, p. 60), whose image plays with our feelings making us doubt whether or not it is a real person who actually stood in front of him (Ramón, 2011, p. 69).

**Figure 9:**
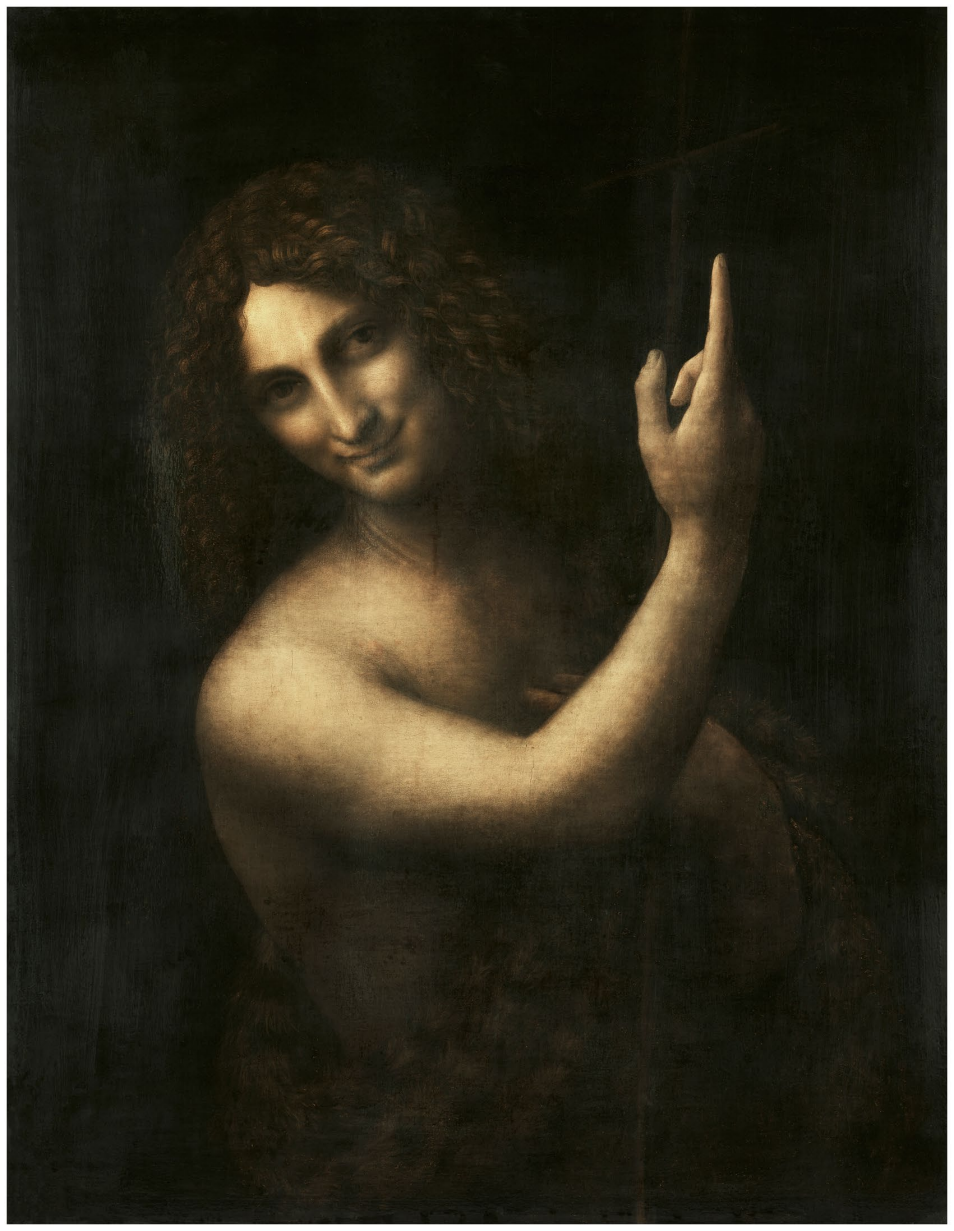
Leonardo da Vinci‘s Saint John the Baptist (1513–1516). Reproduced from rawpixel via Wikimedia Commons, licenced under CC0.

**Figure 10:**
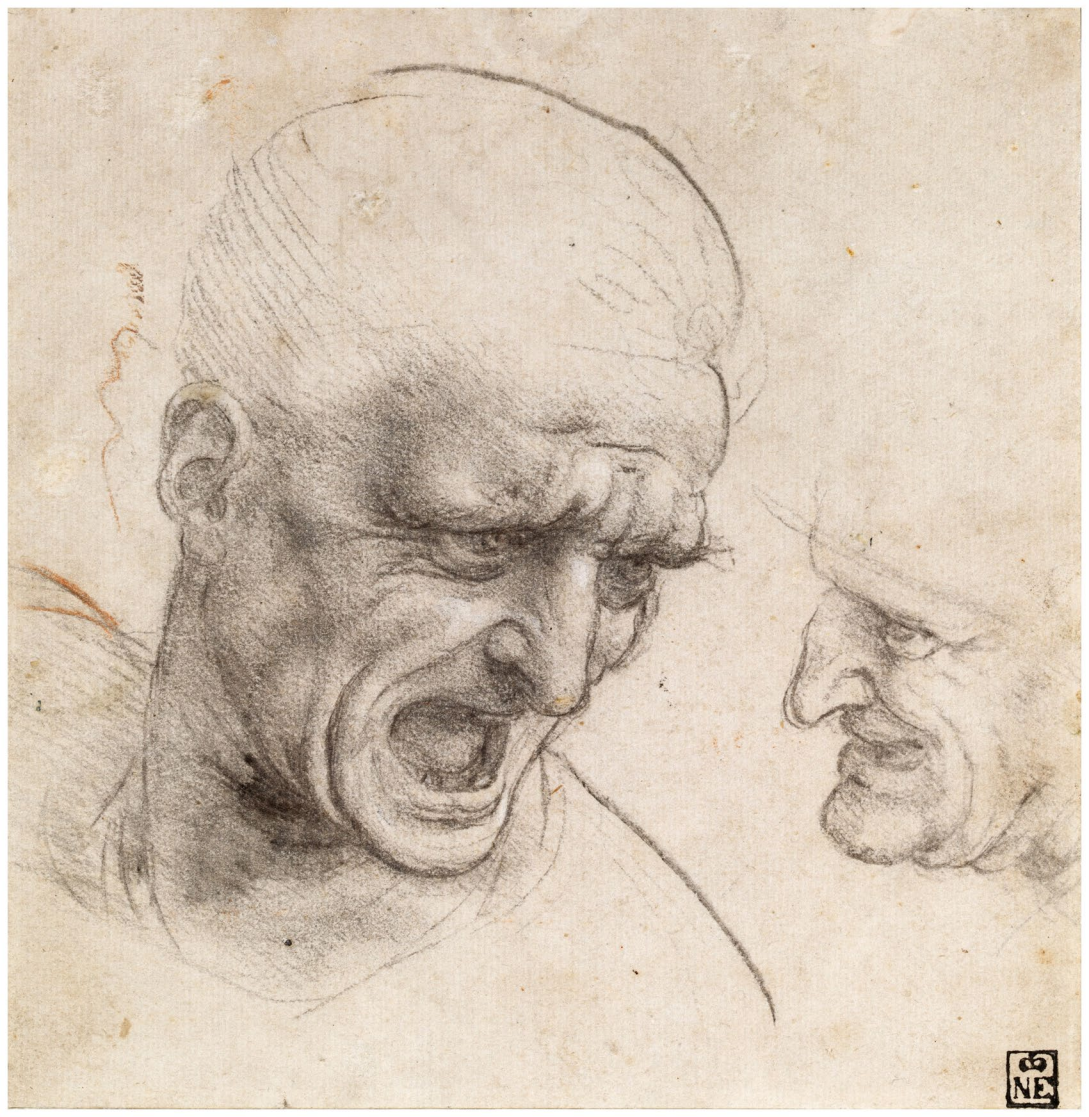
Leonardo da Vinci, Study of Two Warriors’ Heads for the Battle of Anghiari (1775). Public domain, via Wikimedia Commons.

Leonardo’s studies to represent emotions are also related to his notes on the anatomy of the mouth, which include the investigation of the action of the muscles involved in the generation of a smile and show the extensive scientific knowledge that is behind the artistic conception of smiling figures, such as in The Virgin, the Child Jesus and Saint Anne (1503), the Mona Lisa and Saint John the Baptist (1513–1,516, Figure 9), and in screaming figures, as in the studies for the Battle of Anghiari (Figure 10) (Richter, 1977, p. 344). He studied many emotions but although he devoted himself to all the emotions related to the expressions mentioned, he does not speak of them (Pedretti, 1964, p. 133).

After his first biographer, Paolo Giovio (1483–1,552), cited Leonardo as an artist in different fields and also a scholar of optics, anatomy and music, it was Georgio Vasari (1511–1,574) who demonstrated his importance (Pérez de Guzmán, 2003a, p. 18; Heck, 2009, p. 377). However, Leonardo’s work only truly became accessible to the public with the opening of the Louvre in 1800, because, until then, only the Treatise on Painting (a recompilation of his notebooks and leaves put together by Francisco Melzi (1491–1,570)) was known. This collection was kept in the Vatican Library and has been a bestseller since its first edition, in 1651 (Pérez de Guzmán, 2003a, p.17), which was followed by the London edition of 1721 and the Nuremberg edition of 1724; however, the Naples Treatise fills a very large gap as the only complete Italian language version to appear between the Paris edition and the Bologna edition of 1786 (Willette, 2009, p. 147). Overall, between 1,651 and 1898, there have been around 30 editions of the Treatise to date (Müntz, 2005, p. 205).

It is interesting to note that between the 17th century and early 19th century, Leonardo was not at the height of his influence, but over the course of the 19th century, with the emergence of Romanticism and the new spirit of transformation, he was considered a driver of modernity, fascinating diverse individuals like the French poets Theóphile Gautier (1811–1872), who in 1820 wrote in *La Presse* that Leonardo was a painter of the mysterious and compared his paintings to musical notes; Gustave Moreau (1826–1898); and Stéphane Mallarmé (1842–1898) (Pérez de Guzmán, 2003a, p. 18), as well as and Friedrich Wilhelm Nietzsche (1844–1900), who mentioned that Leonardo is one of the magical and enigmatic beings to whom supreme triumph is guaranteed (Sureda, 1998, p. 292).

Regarding modernity, it is interesting to note the syncretism that Leonardo forged between the arts, understanding them as unique and independent, but in close relationship with one another and, in this sense, he managed to change the discriminatory concept that painting maintained among the mechanical arts to integrate it into the liberal arts, thought and beauty (Franqui, 2003, p. 20). It is also important to note the strong spiritual affinity and deep admiration, mainly as a painter, that Goethe had for Leonardo and his universal genius (Adriani, 1978, p. 200; Sureda, 1998, p. 294); for instance, similar to Leonardo, he contemplates nature with analogies in the poem *Epirrhema*. The title is the name given to a part of the chorus in Greek drama, whose significance for the poem is not clear (Goethe, 1966, p. 183), although the point is that we can be ‘outside’ ourselves, observing our behavior, and yet know that we are still somehow inside ourselves too (Goethe, 1966, p.187). This point was eloquently expressed in the poem as follows (Mensch, 2014, p. 86):

“You must, when contemplating nature,

Attend to this, in each and every feature:

There's nothing outside and nothing within,

She’s inside out and outside in.

Thus will you grasp, with no delay,

The holy secret, clear as day.

[…]

No living thing is One, I say,

But always Many.”

## Leonardo, empathy and the expression of his art

“Let your figures have actions appropriated to what they are intended to think or say, and these will be well learnt by imitating the deaf, who by the motion of their hands, eyes, eyebrows, and the whole body, endeavor to express the sentiments of their mind. Do not ridicule the thought of a master without a tongue teaching you an art he does not understand; he will do it better by his expressive motions, than all the rest by their words and examples. Let then the painter, of whatever school, attend well to this maxim, and apply it to the different qualities of the figures he represents, and to the nature of the subject in which they are actors” - Expression and Character, Of Expressive Motions, c. 165 (Da Vinci, 2014, p. 85).

Leonardo was one of the most expressive personalities of the Renaissance. He was mainly an artist, but also a scientist, and knew that study, reflection and technique are preparatory, not creators of the artistic process, since the creation of art requires something more strict and directly divine, which for Leonardo was nature (Gentile, 1978, p. 163). He knew how to observe, reflect, imagine, design, draw and discover physical laws and new instruments of change (Franqui, 2003, p. 19), reacting against formalisms and building his intellectuality and senses based on his own criteria (Pérez de Guzmán, 2003a, p. 17).

He commonly reflected on nature and life on earth, with thoughts surrounded by analogies, from which appears the idea of transmigration of the spirit and the demonstration of its form of analysis and critical exposition (Cortes, 1994, p. 37). Analogical reasoning indicates that constituent parts of an organism can be compared with the other parts and that the process of idealization of a concept points to special ideas that fall into the conceptual question, that is, our analogy of physical organization is more than external (Titchener, 1909, p. 72). In this sense, Leonardo needed to visualize phenomena through their relationships (Pérez de Guzmán, 2003b, p. 23) and often states his analogies paralleling the organism of the earth with the human organism, as illustrated by the following example of his writing (Sacco, 1978, p. 457):

“So that we might say that the earth has a spirit growth; that its flesh is the soil, its bones the arrangement and connection of the rocks of which the mountains are composed, its cartilage the tufa, and its blood the springs of water. The pool of blood which lies around the heart is the ocean, and its breathing, and the increase and decrease of the blood in the pulses, is represented in the earth by the flow and ebb of the sea; and the heat of the spirit of the world is the fire which pervades the earth, and the seat of the vegetative soul is in the fires, which in many parts of the earth find vent in baths and mines of sulfur, and in volcanoes” (Code Leicester, fol. 34 r).

According to the dictionary of the Royal Spanish Academy (Real Academia Española, 2022), analogy is the reasoning based on the existence of similar attributes in different beings. In fact, the sense of harmony between the different phenomena of the planet, between man and the Universe, between the micro- and the macrocosmos and the conception of the world as an immense network already existed since antiquity and remains one of the major concerns of contemporary thought (Pérez de Guzmán, 2003b, p. 23). We often end up simply depicting a character or external object rather than identifying its constitutive factors, that is, the relationship of our mind with what we see is much more than merely an exercise in pointing something out, but a necessary analysis of implication between the parts (Titchener, 1909, p. 70).

The analogies used by Leonardo meant that his drawings represented a unique scientific research method, showing that the image is not only the complementary illustration of a text, but the vehicle of a technical thought, a mental discourse (Cortes, 1994, p. 37) — the result of a developed knowledge about the truth of the things represented (Ramón, 2011, p. 69). Lipps asserts that however true it may be to say that the question of reality or non-reality does not in any way affect the essence of the work of art as an object of aesthetic contemplation, it is no less true that —not in all the arts, but in a certain class of them— a certain relationship with reality is necessary (1924, p. 59), as with Leonardo’s drawings. In this regard, Titchener believed that the picture is combined with an empathic attitude and all such feelings of if, and why, and nevertheless, and therefore, normally take the form of a kind of mimicry or motor empathy (1909, p. 185). The feeling is acted out even though it may be fleeting, or it may be relatively stable; whichever it is, there is not the slightest doubt of its kinesthetic character (Titchener, 1909, p. 185).

From da Vinci’s point of view, visual language had supremacy over verbal language and was key for figurative communication, especially the equivalence of mechanical arts with liberal arts, as advocated in the Treatise on Painting (Ruiz García, 2011, p. 225). Leonardo’s exaltation of the importance of the eyes, which he called windows of the soul, has extraordinary anthropological value because it corresponds to the historical moment in which he lived, the transition from the medieval to the modern world, marked by the influence of visual perception on the senses of hearing and smell in the representation of the human being and its relationship with the natural world (Vecce, 2003, p. 61). Other well-known figures in the 20th century were supportive of this view, including Virginia Woolf (1882–1941), who wrote in *Books and Portraits* (Woolf, 1977, p. 36):

“The heather is not much, and the rock is not much; but the heather and the rock, discerned in their living expressional relationship by the poetic eye, are very much indeed — a beauty which is living with the life of man, and therefore inexhaustible … but true poets and artists know that this power of visual synthesis can only be exercised, in the present state of our faculties, in a very limited way; hence, there is generally, in the landscapes and descriptions of real genius, a great simplicity in and apparent jealousy of their subjects, strikingly in contrast with the works of those who fancy that they are describing when they are only cataloging.”

**Figure 11:**
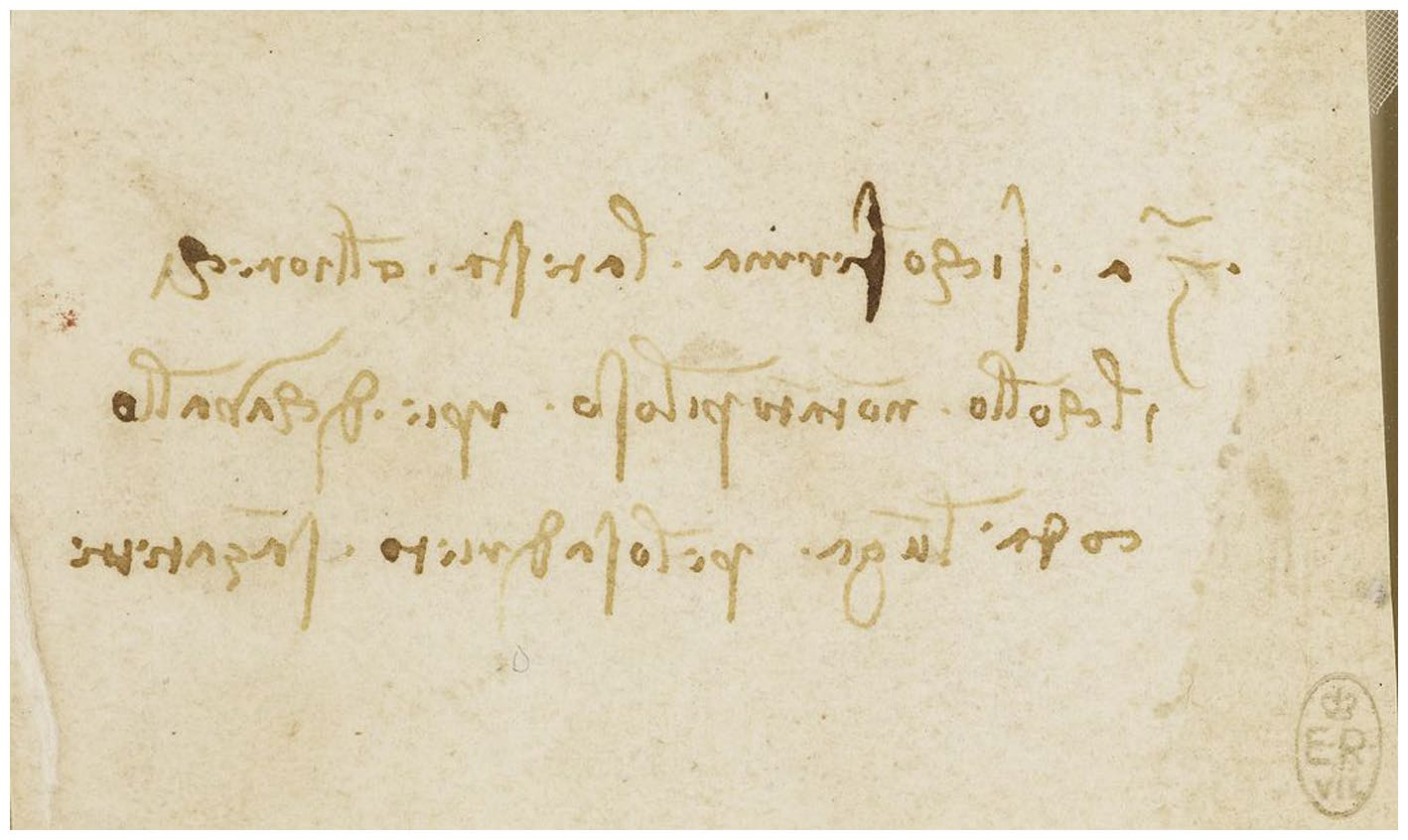
Notes on the appearance of horses c.1490. Some written notes in Leonardo’s hand. Pen and ink | 5.0 × 8.1 cm (sheet of paper). Public domain, via Wikimedia Commons.

For Leonardo, painting had the necessary signals to express different languages and, although he recognizes the presence of writing, he considers it as a form of drawing when commenting that writers draw with the pen what is found in their mind (Vecce, 2003, p. 61). There is no better way to verify this fact than to contemplate his signature, which denotes a great vital dynamism, an imaginative mind, a taste for originality and an enviable aesthetic sense (Ruiz García, 2011, p. 199; see Leonardo’s notes in Figure 11).

**Figure 12:**
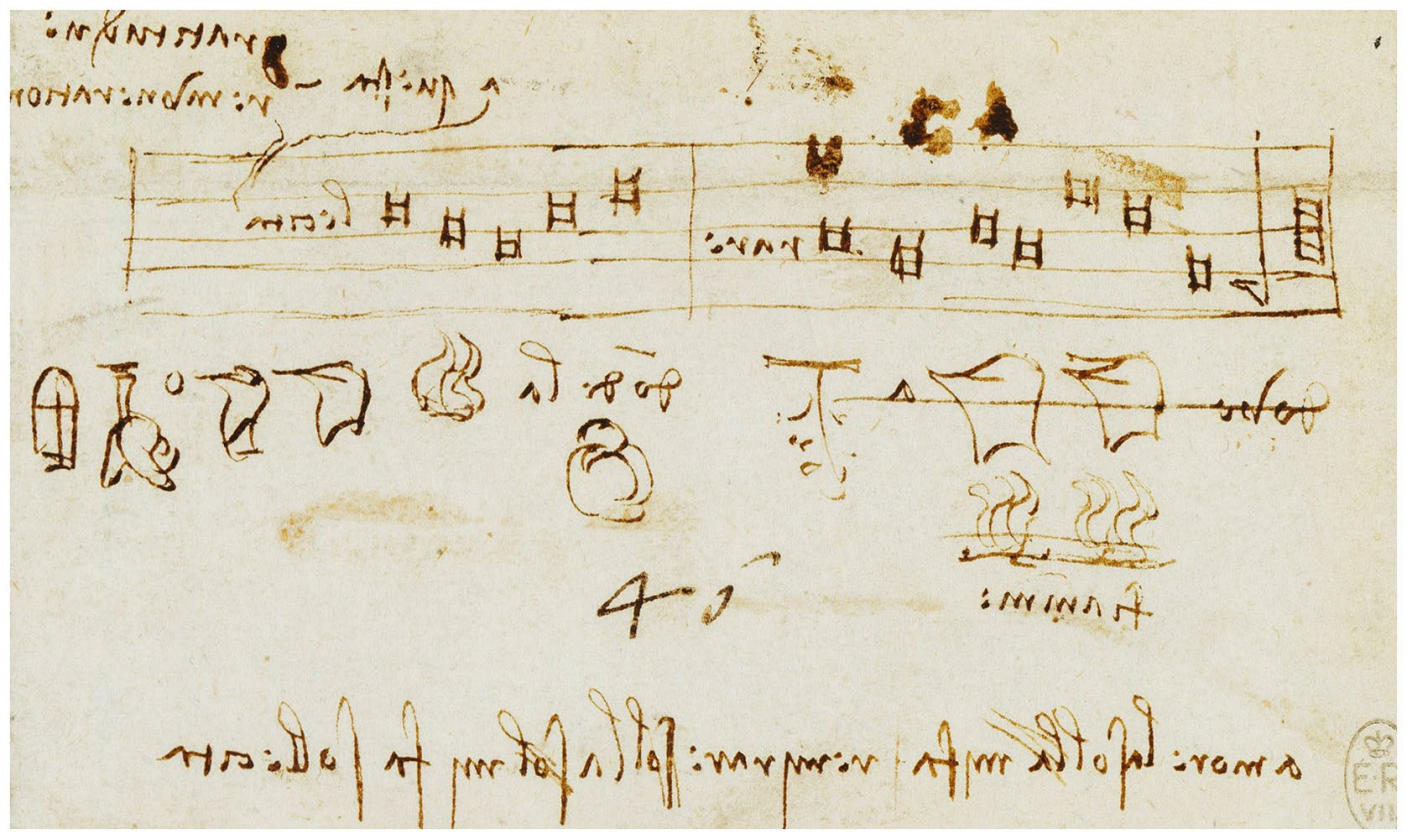
Leonardo da Vinci, Pictographs c.1487–90. A scrap of puzzle-writing, the first line being made of musical notes to be interpreted on the la, sol, mi, fa system. In the middle, the beginning of various attempts to put into pictographs the sentence previously given. Pen and ink | 6.0 × 10.3 cm (sheet of paper). Public domain, via Wikimedia Commons.

The relationship between image and word is one of the fundamental elements in Leonardo’s work through which he plays with drawings and writings with genius and innovation, freely and without the correct construction of language, metric or cultural traditions (Pérez de Guzmán, 2003b, p. 28; see Leonardo’s pictographs in Figure 12). Leonardo believed that to produce a result by means of an instrument to drawn or write does not allow does not allow yourself to complicate it by introducing many subsidiary parts, but rather results in following the briefest way possible, without acting as those who do not know how to express a thing in its own proper vocabulary and proceed by a method with great prolixity and confusion (Richter, 1977, p. 245).

The point of Leonardo’s drawings was to make an object immediately visible, with little explanation required, that is, to highlight the relationship between who sends the message and who receives it — an approach that marked the beginning of modern science, and one that Leonardo employed to convey the meaning of his games between figures and words (Vecce, 2003, p. 69). As a matter of fact, mental constitution is widely varied, and the meaning response of a mind of a certain constitution varies widely under varying circumstances; all conscious meaning is carried either by total kinesthetic attitude or by words — as well as by all sorts of sensational and imaginal processes (Titchener, 1909, p. 178).

Leonardo’s drawings show his speculative thinking — and imagination was undoubtedly utmost in his mind, as he used iconic elements as substitutes for corresponding words, in line with the notion of the predominance of figurative resource over linguistic resource (Ruiz García, 2011, p. 216). The angel prototype that Leonardo painted in Baptism of Christ and the teenager in Adoration of the Magi are examples in which his imaginative soul guided his hands in forming an image with its own characteristics (Steinitz, 1960, p. 118). Thus, one of the elements of Leonardo’s research, in addition to his artistic ability, and search for scientific knowledge, was fantasy or creative imagination, always linked to the intellectual understanding of nature (Capra, 2011, p. 63). He was clearly convinced that fantasy was an imaginative extension of rational thought rather than a negation of it (Kemp, 2003, p. 147). Eugène Müntz, a French art historian (1845–1902), declared that no artist was so independent as to interpret both imagination and creation (Steinitz, 1960, p. 118).

Leonardo’s drawings and writings are the result of rigorous and systematic observation accompanied by isolated moments of disorganized expression; in other words, they are eminently mental constructions in which calculated synthesis captures the sensitive information of what is observed to shape and organize his work (Bongioanni, 1978, p. 184). Over the centuries, efforts have been made to explain and codify the outward manifestations of character, thoughts, and emotions, which Leonardo called *il concetto dell’anima* - the intention of the mind (Kemp and Wallace, 2000, p. 16). In this sense, he believed that the most important thing in painting the human figure was to represent mental states and emotions, since the expression of the human spirit through art was the artist’s greatest aspiration (Capra, 2011, p. 33). Moreover, a true painter should know the truth of the things he represents to make it possible to see the nature of what is expressed by art (Ramón, 2011. p. 26). For Leonardo, discovering nature meant finding an order of connection that the discoverer is a part of and in which he identifies the sense of his presence in the idealized system (Bongioanni, 1978, p. 184).

**Figure 13:**
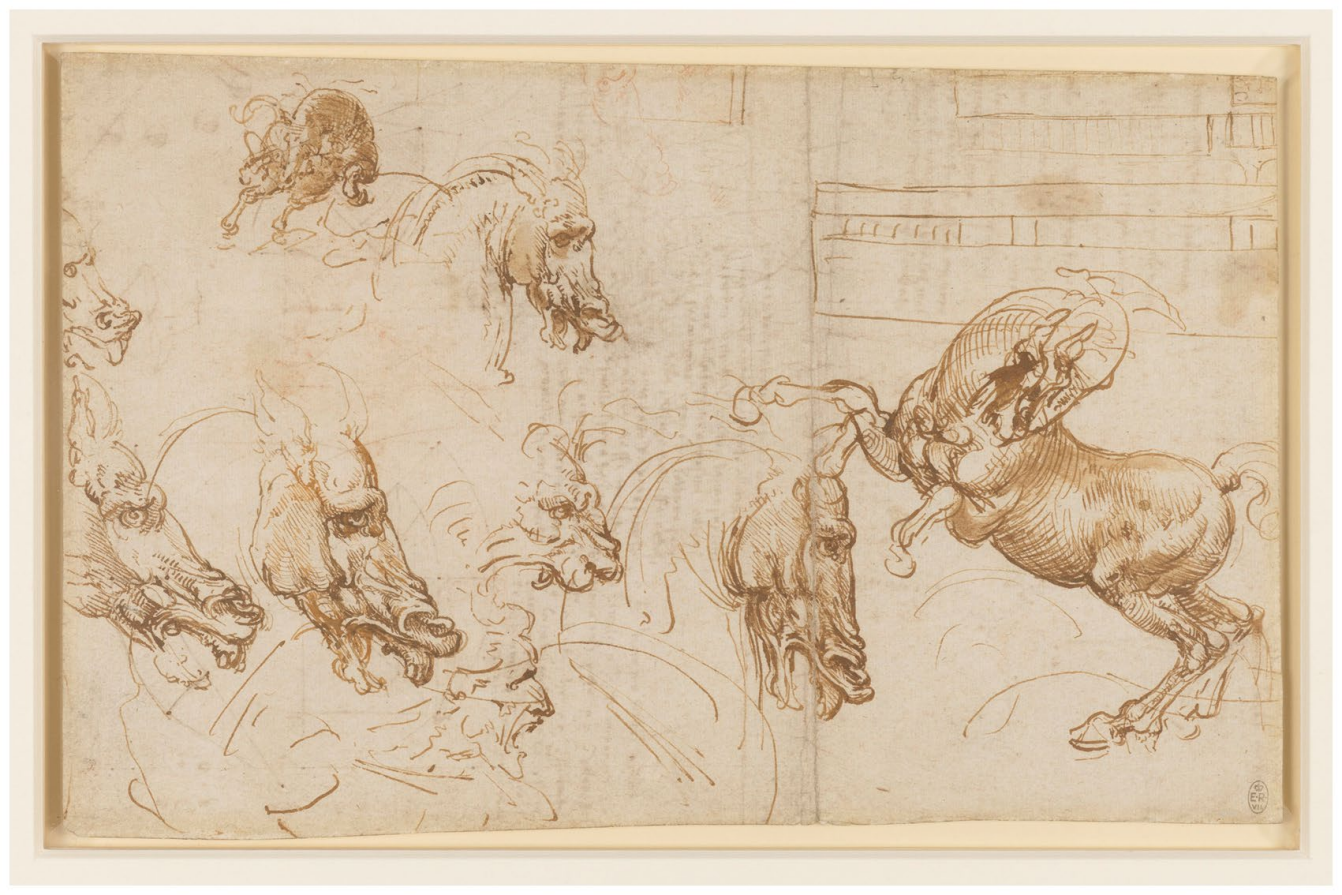
Leonardo da Vinci, A rearing horse, and heads of horses, a lion and a man. c.1503–4. Pen and ink, wash, a little red chalk. | 19.6 × 30.8 cm (sheet of paper). Public domain, via Wikimedia Commons.

**Figure 14:**
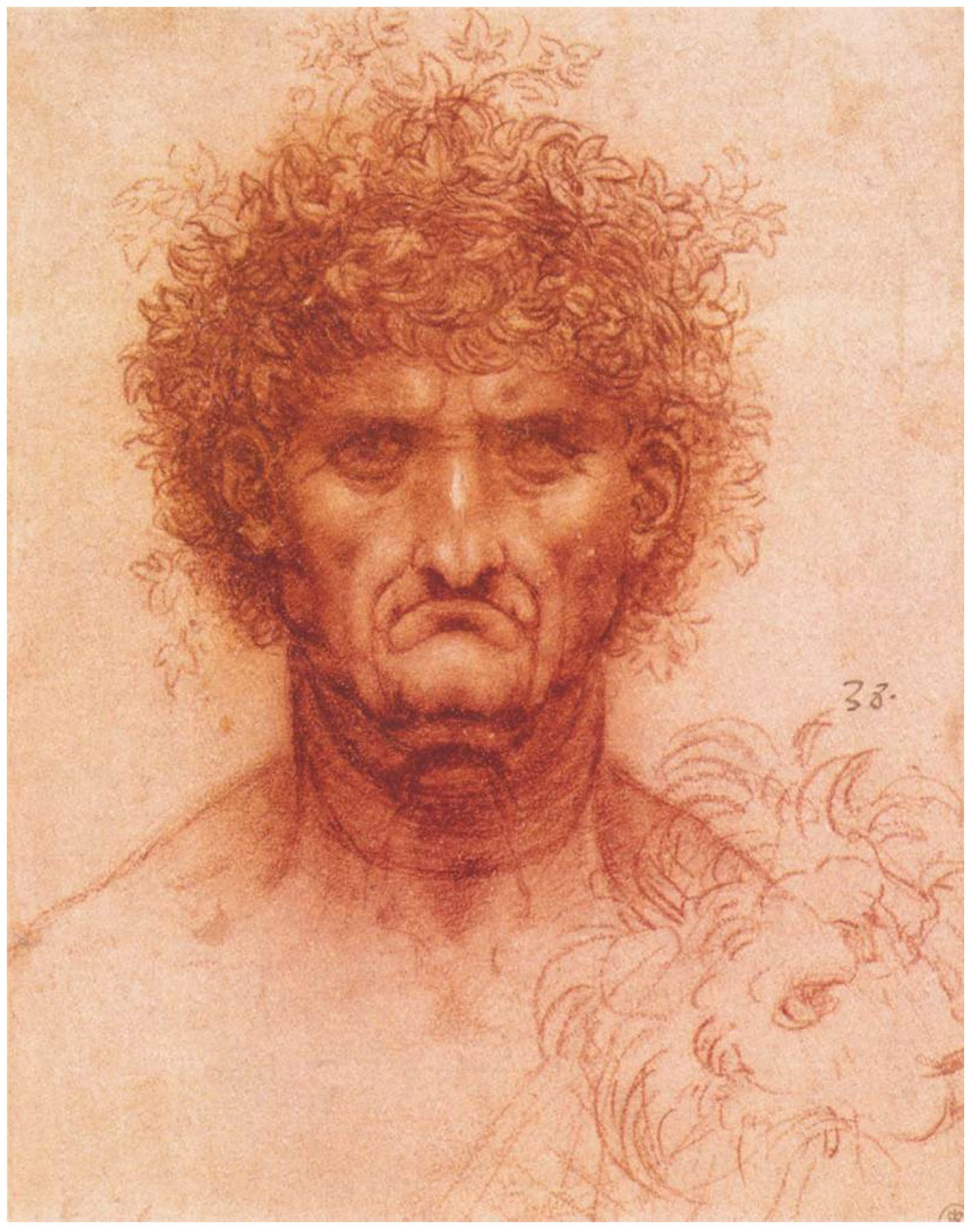
Leonardo da Vinci, The bust of a man, and the head of a lion c. 1,510. Red chalk, touches of white chalk, on orange-red prepared paper | 18.3 × 13.6 cm (sheet of paper) Public domain, via Wikimedia Commons.

Leonardo recognized that for the painter to understand the structure of a figure in order to give expression to his spirit, it was fundamental to study —in different species, ages and sexes (Figures 13, 14)— the different body constitutions; anatomy; relations and proportions; attitudes; movements; and mimetic elements (Biaggi, 1978, p. 439). In this way, it is possible to communicate feelings, impressions and ideas and reproduce reality with the greatest accuracy (Pedretti, 2003, p. 94). For him, the painter ought to study methodically and leave nothing unmemorized and he must observe how limbs and joints vary from one animal to another (Richter, 1977, p. 101).

**Figure 15:**
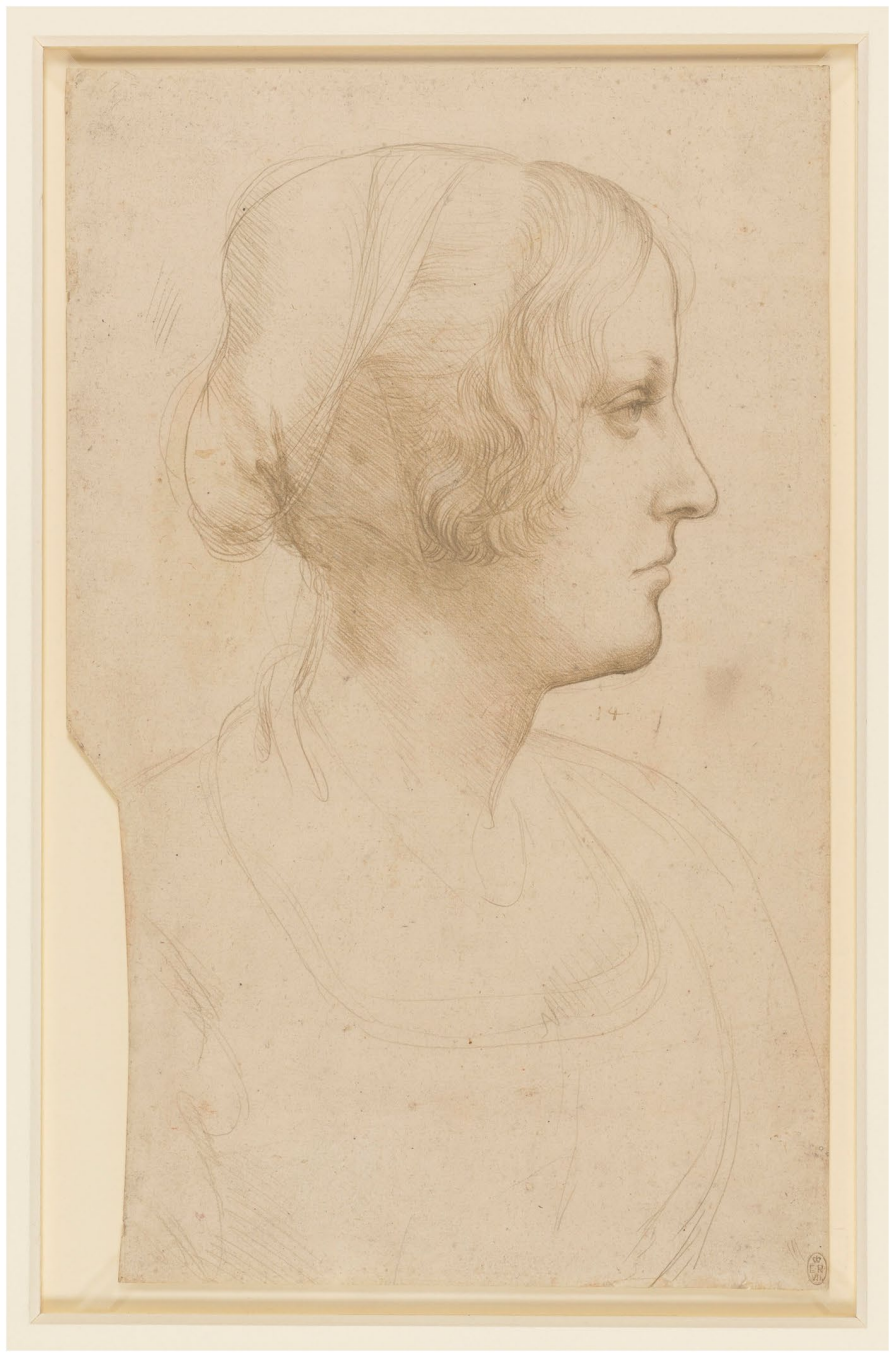
Leonardo da Vinci, Portrait of a young woman in profile c.1490 (RCIN 912505). Public domain, via Wikimedia Commons.

The various aesthetic images, as possible alternatives of logical meaning, often share their functions with the sensations of movement (Titchener, 1909, p. 21). Thus, whenever one feels oneself from the observation of an attitude or gesture in an external object, this projection of the object itself is the very feeling triggered by an inner effort (Lipps, 1924, p. 7). At a basic level, *einfühlung* points to feeling of belonging in the world; likewise, it refers to an immediate and intersubjective relational experience —a kinetic inner imitation— a mirroring of the expression observed that carries affectively perceived sensorimotor impressions (Stamatopoulou, 2018, p. 170). In this sense, Leonardo possessed that rare combination of vitality, strength and delicacy which only a few of the greatest draftsmen have achieved (Kemp, 2003, p. 33; also see Figure 15) and that provides the observer with a voluntary and conscious activity of fantasy and contemplation of objects. Furthermore, the richness of the artist’s fantasy is, therefore, the richness of that which happens in the work, or richness of its content, concretely, of moments of inner vitality expressed in the work (Lipps, 1924, p. 95). In Expression and Character, from Of the Variety of Faces (c. 170), Leonardo states (2014, p. 86):

“The countenances of your figures should be expressive of their different situations: men at work, at rest, weeping, laughing, crying out, in fear, or joy, and the like. The attitudes also, and all the members, ought to correspond with the sentiment expressed in the faces.”

Leonardo and Vesalius were the pioneers in demonstrating what was possible with anatomical illustrations, from which artists developed aspirations to encourage the viewer to become a witness of what he saw (Kemp and Wallace, 2000, p. 33). In this regard, he affirmed that the painted figures should be created so that the observer could easily know, through the movements and attitude of the figures, the mental situation of the creator of the narrative and the meaning of his intentions (Da Vinci, 2013, p. 121, c. 294), since an experiencer must empathize with an observer in order to think, understand and communicate as he does (Titchener, 1909, p. 185). After all, there is no doubt that the expression of emotions and feelings are an integral part of who we are, personally and socially (Damasio, 2003, p. 247).

Leonardo’s painting is not understood at first sight; a mental process is required on the part of the observer to reconstruct and appreciate all its richness of expression, movement, gesture and aesthetic configuration. The spectator must create unity, just as the ear perceives a series of notes and silences that the listener’s brain configures as something invisible (Pérez de Guzmán, 2003c, p. 117). As with other brain functions, it seems that we have to learn to appreciate art, sometimes by a cultural or a perceptual transformation (DeFelipe, 2014, p. 78). The harmony of his work is not born directly from the figure, but from an infinity of details that, together, constitute the work. As Leonardo put it, details make perfection, and perfection is not a detail (Pita, 2019, p. 39).

Finally, thanks to the development of social neuroscience, which combines classical cognitive neuroscience and social psychology, using multi-level and multidisciplinary approaches, the neural basis of empathy is being unraveled (e.g., see Bernhardt and Singer, 2012). Furthermore, greater links between neuroscience and the humanity field can be seen represented by enhanced communication encompassing neuroscience and the humanities, clear conceptual overlap between both fields, and new actionable outcomes (Carew and Ramaswami, 2020). Who could have imagined that Leonardo’s dream of explaining artistic perception with a mechanistic model would become a feasible scientific goal as it is presently?

## Author contributions

SM: Investigation, Writing – original draft, Conceptualization, Writing – review & editing. JD: Supervision, Writing – review & editing, Funding acquisition.
